# Phase-free local ancestry inference mitigates the impact of switch errors on phase-based methods

**DOI:** 10.1093/g3journal/jkaf122

**Published:** 2025-06-05

**Authors:** Siddharth Avadhanam, Amy L Williams

**Affiliations:** Department of Computational Biology, Cornell University, Ithaca, NY 14853, USA; Department of Computational Biology, Cornell University, Ithaca, NY 14853, USA; Department of Computer Science, Brigham Young University, Provo, UT 84602, USA

**Keywords:** local ancestry inference, admixture, phase, haplotypes, ancestry

## Abstract

Local ancestry inference is an indispensable component of a variety of analyses in medical and population genetics, from admixture mapping to characterizing demographic history. However, the accuracy of local ancestry inference depends on a number of factors such as phase quality (for phase-based local ancestry inference methods) and time since admixture. Here, we present an empirical analysis of four local ancestry inference methods using simulated individuals of mixed African and European ancestry, examining the impact of variable phase quality and a range of demographic scenarios. We find that regardless of phasing options, calls from local ancestry inference methods that operate on unphased genotypes (phase-free local ancestry inference) have 2.6–4.6% higher Pearson correlation with the ground truth than methods that operate on phased genotypes (phase-based local ancestry inference). Applying the TRACTOR phase correction algorithm led to modest improvements in phase-based local ancestry inference, but despite this, the Pearson correlation of phase-free local ancestry inference remains 2.4–3.8% higher than phase-corrected phase-based approaches (considering the best-performing methods in each category). Further, analyzing perfectly phased data yields accuracies for the phase-based local ancestry inference methods that are only slightly inferior to those of HAPMIX. Phase-free and phase-based local ancestry inference accuracy differences can dramatically impact downstream analyses: estimates of the time since admixture using phase-based local ancestry inference tracts are upwardly biased by ≈10 generations using our highest quality statistically phased data but have virtually no bias using phase-free local ancestry inference calls. This study underscores the strong dependence of phase-based local ancestry inference accuracy on phase quality and highlights the merits of local ancestry inference approaches that analyze unphased genetic data.

## Teaser

Inferring the source population of genetic markers in an individual, or local ancestry inference (LAI), is important for numerous applications in genetics. However, few independent analyses of existing LAI methods’ performance have been done, and while the impact of phase uncertainty on LAI is broadly appreciated, this relationship has not been examined in detail. Avadhanam and Williams’ work aims to bridge this gap and their findings indicate that in datasets with thousands of individuals, phase-free approaches outperform phase-based approaches.

## Introduction

The problem of inferring the ancestral population of each locus in an admixed individual’s genome, or local ancestry inference (LAI), has received attention for well over a decade (Hoggart *et al.* 2004; Montana and Pritchard 2004; Patterson *et al.* 2004; Sankararaman *et al.* 2008; Price *et al.* 2009; Baran *et al.* 2012; Maples *et al.* 2013; Guan 2014; Browning *et al.* 2023), and a variety of LAI approaches now exist. Early LAI methods only analyzed markers that are not in linkage disequilibrium (LD) (Hoggart *et al.* 2004; Montana and Pritchard 2004; Patterson *et al.* 2004), thus reducing the information available for inference while greatly simplifying the modeling. The advent of the Li and Stephens haplotype model (Li and Stephens 2003), which accounts for LD, enabled the development of several LAI methods that leverage all available marker data. HAPMIX (Price *et al.* 2009) was one of the first approaches to exploit this rich information, which yielded dramatic improvements in both the accuracy and resolution of LAI compared to earlier methods. Subsequent methods that allow for multiple source populations or have improved runtime scaling have been developed (Baran *et al.* 2012; Maples *et al.* 2013; Guan 2014; Browning *et al.* 2023), with many recent LAI methods requiring that the admixed individuals be phased prior to analysis (Maples *et al.* 2013; Browning *et al.* 2023). On the other hand, methods like HAPMIX, LAMP-LD (Baran *et al.* 2012), and ELAI (Guan 2014) analyze unphased genotypes and output inferred local ancestry in an unphased manner.

Local ancestry calls have wide-ranging use in both medical and population genetics. Admixture mapping (Winkler *et al.* 2010; Shriner 2017) detects trait-associated loci by exploiting the fact that inferred local ancestry tracts are a proxy for variants not directly tested in a study and for a large number of haplotypic backgrounds from the same population. Moreover, methods exist for increasing the power of genome-wide association studies (Pasaniuc *et al.* 2011; Atkinson *et al.* 2021) and for improving heritability estimates and mapping in eQTL studies (Zhong *et al.* 2019) by including local ancestry calls. At the same time, population genetic analyses have used LAI calls to characterize demographic patterns among admixed groups, detecting geographic trends that reflect historical migrations (Bryc *et al.* 2015), and have inferred times since admixture using local ancestry tract lengths (Gravel 2012).

Phasing errors, or mis-assignment of alleles to haplotypes (herein measured as switch errors) can confound LAI by introducing a short tract of a different ancestry onto a haplotype (in cases of two nearby switch errors) or by prematurely ending a tract. Besides merely switching the ancestry assignment to the opposite haplotype, switch errors decrease the quality of LAI calls in part because accurately detecting tract boundaries is difficult, and short, incorrectly phased tracts may not be detected. Furthermore, LAI switch errors can have important consequences for inferring admixture demography in that they alter observed tract lengths (Gravel 2012). Phase-based LAI methods sometimes attempt to account for phasing errors, as in RFMix, which jointly models switch errors and local ancestry (Maples *et al.* 2013). By contrast, phase-free LAI methods do not read phase information for the target samples and are, therefore, unaffected by switch errors.

Recently developed statistical phasing methods can scale to thousands or even millions of individuals (Browning *et al.* 2021; Hofmeister *et al.* 2023), and when analyzing the UK Biobank or 23andMe datasets, can correctly phase even entire chromosomes for many samples (Williams *et al.* 2024; ignoring single site errors). Even so, such tremendous phase quality is only achievable given either family data or very large sample sizes (since statistical phasing accuracy is a function of sample size Browning and Browning 2011). Phase accuracy is also a function of the number of genotyped variants (Browning and Browning 2011) and genotyping accuracy. Sample sizes, variant density, and genotype accuracy are often lower when analyzing nonhuman data, particularly in nonmodel organism studies. As such, it is beneficial to consider the impact of phase quality on LAI, noting that phase accuracies that would be considered low in human studies may be representative or even high when analyzing nonhuman data.

Given the many applications of LAI and an appreciation of the associated phasing issues, characterizing the performance of LAI methods is important; yet, many comparisons are in the papers that describe new methods, with relatively few independent analyses. One recent LAI benchmarking study included a range of LAI methods and is complementary to our work in that it does not analyze the impact of phasing errors on the tools (Schubert *et al.* 2020).

Here, we present a comparison of four LAI methods using genome-wide data for simulated individuals of mixed African and European ancestry. To appropriately model phasing error, we converted the simulated haplotypes into diploid genotypes and phased these using a range of sample sizes, thus accounting for and quantifying the impact of phasing errors on LAI. Moreover, we considered the impact of demography—genome-wide ancestry fraction and time since admixture—as well as phasing choice with respect to the LAI reference panels on LAI accuracy.

## Materials and methods

**Fig. 1.**
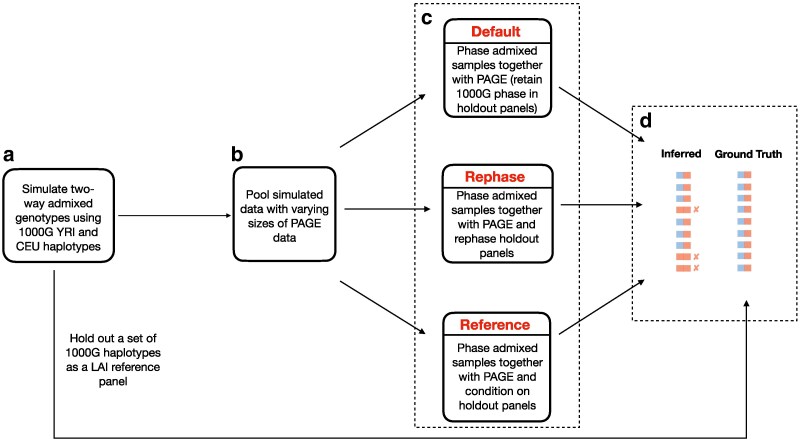
Flowchart depicting our simulation and phasing pipeline. In step a), we simulated two-way admixed individuals under different settings of *T* and *p*; in step b), we pooled the simulated data from a) with small, medium, and large sample sizes from the PAGE data; and in step c), we applied three different panel phasing strategies (see Varying simulation phase quality and panel phasing strategies in Materials and methods). Finally in step d), we inferred local ancestry on the output from c) and compared the resulting inferred calls with the ground truth using Pearson correlation.

To systematically investigate the effect of various demographic parameters and phasing options on LAI accuracy, we developed a pipeline (Fig. 1) that: (1) simulates genotypes of mixed African and European ancestry with African ancestry proportion p∈{0.2,0.5,0.8} and time since admixture T∈{5,6,7} (and separately T∈{5,10,15}); and (2) phases these genotypes using three different sample sizes (noting that sample size substantially impacts phase quality Browning and Browning 2011). At the same time, we varied phasing with respect to the unadmixed reference panels by three different panel phasing strategies (see below). To further quantify how much phase quality affects LAI accuracy, we also analyzed perfectly phased haplotypes as generated by simulation.

The LAI methods we benchmarked fall into two categories: phase-free, which take unphased data as input and output unphased local ancestry calls, and phase-based, which require phased haplotypes and output phased local ancestry calls. In the phase-free category, we applied HAPMIX (Price *et al.* 2009; version 2) and LAMP-LD (Baran *et al.* 2012; version 1.1), and in the phase-based category we ran RFMix (Maples *et al.* 2013; version 2), and FLARE (Browning *et al.* 2023; version 0.3.0). Notable omissions from our list of LAI methods include LOTER (Dias-Alves *et al.* 2018), MOSAIC (Salter-Townshend and Myers 2019), ELAI (Guan 2014), and MULTIMIX (Churchhouse and Marchini 2013), which have comparable overall accuracy to the approaches we analyzed (Maples *et al.* 2013; Schubert *et al.* 2020). The different phasing options we applied reflect workflows that could occur under variable real-world constraints—namely, access to admixed datasets of different sizes, choice of panel-based phasing strategy, and choice of LAI method (which may itself be constrained by phase quality). For example, a user that has few admixed samples from a population of interest might phase them jointly and retain the phase supplied by (for example) the 1,000 Genomes Project (1,000 G) (1000 Genomes Project Consortium *et al.* 2015) for the unadmixed reference haplotypes (i.e. rather than rephasing this panel—see below). There are also several statistical phasing tools to choose from (Loh *et al.* 2016; Delaneau *et al.* 2019; Browning *et al.* 2021), and in this study we used SHAPEIT4 (Delaneau *et al.* 2019). Alternatively, the phasing step may not be necessary if a user applies a phase-free LAI method such as HAPMIX or LAMP-LD.

We evaluated the LAI accuracies by measuring the Pearson correlation (*R*) between the inferred and simulated local ancestry calls (the latter are the ground truth assignments) across all markers. We calculated this correlation using unphased (i.e. diploid) local ancestry calls for two reasons. First, measuring accuracy in a phase-based way is complicated by the presence of switch errors that are introduced upstream of the LAI method. Second, ignoring phase ensures a uniform metric of comparison between phase-based and phase-free LAI methods.

Together with these accuracy calculations, we evaluated the LAI methods’ performance for estimating time since admixture using an adapted version of a Markovian model described by Gravel (2012). We applied this model directly on the output of the phase-based method FLARE and indirectly using PAPI (Avadhanam and Williams 2022) on the output of the phase-free method HAPMIX.

### Data-efficient simulation of admixed genotypes

In recent work (Avadhanam and Williams 2022), we described a pipeline for simulating admixed individuals that first uses Ped-sim (Caballero *et al.* 2019) to generate crossovers in a fixed pedigree, and then uses admix-simu (Williams 2016) to sample haplotype segments at each crossover breakpoint. The latter draws each segment from the same population as the unadmixed ancestor that transmitted it, with the advantage (versus simply using Ped-sim) of requiring far fewer unadmixed individuals to simulate each person. As an example, unmodified, Ped-sim requires 128 founders to simulate one admixed individual when T=7. Furthermore, these founders must be excluded from any subsequent simulation in order to produce unrelated samples. Our pipeline uses far fewer founders, with the requisite number being independent of *T* (Avadhanam and Williams 2022). We employed a pedigree topology containing the admixed individual in the most recent generation (generation 0) and all that person’s ancestors up to generation *T* included (i.e. with $2^{T}$ founders in the oldest generation). The pipeline also uses the parameter *p* to specify the proportion of founders of African ancestry (Avadhanam and Williams 2022).

In order to make maximal use of the unadmixed 1,000 G individuals and reserve some as LAI reference panels, we simulated by dividing the unadmixed data into three batches, each composed of a nonoverlapping set of 35 YRI and 31 CEU individuals (out of 107 YRI and 95 CEU samples). We mapped these 3 batches arbitrarily to the 3 settings of p∈{0.2,0.5,0.8} and simulated admixed individuals from that assigned founder batch for all 3 settings of *T*. To better represent the variance from sampling crossover breakpoints and founder haplotype segments, we simulated 3 replicates of 30 admixed individuals for each of the nine settings of *T* and *p* (for a total of 90 admixed individuals). We used the phase supplied by 1,000 G for these simulations and excluded all trio/duo children. This process ensures that each batch has a corresponding nonoverlapping holdout set of 72 YRI and 64 CEU individuals for use as LAI reference panels.

Following these simulations, for each replicate, we merged the 30 simulated samples with additional admixed individuals of three different sample sizes (small, medium, and large) and phased these using three different panel phasing strategies (see the next subsection and Fig. 1). Considering all sample sizes and phasing strategies, this yielded a total of nine phasing conditions analyzed for each setting of *p* and *T*. A benefit of this is that every comparison between phasing sample size and panel strategy operate on the same simulated individuals, thus automatically controlling for the noise generated by the simulation of admixed genotypes. In other words, while comparisons between different values of *p* and/or *T* analyze different simulated admixed samples, comparisons between different phasing sample sizes or panel phasing strategies (but the same *p* and *T*) consider the exact same simulated data.

### Varying simulation phase quality and panel phasing strategies

We induced varying degrees of phase quality by pooling the simulated admixed individuals with different numbers of individuals from the BioMe Biobank subset of the Population Architecture using Genomics and Epidemiology (PAGE II; Wojcik *et al.* 2017; Wojcik *et al.* 2019) study and phasing them jointly using SHAPEIT4 (Delaneau *et al.* 2019; see below and Fig. 1). To that end, we ascertained two-way admixed PAGE individuals of mixed African and European ancestry by following a procedure that we employed in earlier work (Avadhanam and Williams 2022). We first ran ADMIXTURE (Alexander *et al.* 2009) with K=5 on the PAGE samples merged with 176 HapMap trio parents evenly split between the CEU and YRI populations; this allowed us to determine which of the *K* components corresponded to African and European ancestry. We then selected individuals that have (1) ≥5% African ancestry; (2) ≥5% European ancestry; and (3) the sum of these two ancestries ≥99.5%; this yielded 5,786 PAGE samples. We then randomly sampled 5,780 of these PAGE individuals to use as the “large” sample size, 2,890 for the “medium” sample size, and 580 for the “small” sample size. We verified that the phasing error generally correlated inversely with sample size by measuring switch error rates using vcftools with the --diff-switch-error flag, where we took the phase output by the simulator to be the ground truth (see Results).

Besides varying the sample size, we applied three different panel-based phasing strategies: (1) the “default” phasing strategy, where we retained the original 1,000 G phase for the panels; (2) the “reference” phasing strategy, where we use the --reference option so that SHAPEIT4 conditions on the panel haplotypes when phasing the admixed samples (this is recommended when using such a panel for downstream analyses such as imputation or LAI); and (3) the “rephase” strategy, where we pooled the reference panels with the admixed genotypes and retrieved the resulting rephased haplotypes for use as panels for LAI. The latter two strategies help make the admixed individuals’ phase more consistent with those of the reference panel, which contrasts with the default strategy, where the reference haplotypes have the phase present in the 1,000 G data—which were generated independent of the admixed individuals.

### Filtering and processing the PAGE data

The data we used for this work is a merging of the PAGE and 1,000 G data that we used previously. Full details of the merging process and quality control filters we applied are available in our earlier work (Avadhanam and Williams 2022), and we provide a brief summary here. The key steps of the pipeline are filtering SNPs in the PAGE dataset and intersecting these filtered data with the 1,000 G dataset to obtain a common set of SNPs. We filtered the PAGE SNPs using the quality control report distributed with the dataset. This applies a composite filter including those for, among others, Hardy–Weinberg equilibrium, sites with discordant calls in duplicated samples, and those with Mendelian errors. We further ensured there were no allele coding inconsistencies between the two datasets by recoding the PAGE data to the forward strand, filtering out A/T and C/G SNPs, and applying an allele frequency difference test filter. These steps yielded 494,219 markers common to both the datsets, which we used for all analyses.

### Performing LAI and measuring accuracy

After phasing with SHAPEIT4, we passed the datasets as input to each of the LAI methods (in the case of phase-free LAI, we erased the phase) and we used 1,000 G YRI and CEU haplotypes as LAI reference panels. As described above, we ensured that in all cases the LAI reference panels and the set of individuals that we used as simulation founders were disjoint. We ran the LAI methods with default settings (except for FLARE where we set min-mac=0 and min-maf=0) and on each chromosome separately.

We compared the inferred local ancestry calls with the ground truth by representing all markers sequentially (across all 22 chromosomes) in vectors $A_{\inf}$ and $A_{\mathrm{true}}$ whose elements a∈{0,1,2} represent the number of European haplotypes at a site. The Pearson correlation coefficient (*R*) between $A_{\inf}$ and $A_{\mathrm{true}}$ then gives a measure of performance for each simulation setting and LAI method used. For HAPMIX, which outputs a posterior probability of each ancestry state at each marker (i.e. P(a) for a∈{0,1,2}), we first calculate $E[A_{\inf}]$ before computing the Pearson correlation coefficient between $E[A_{\inf}]$ and $A_{\mathrm{true}}$. The elements of $E[A_{\inf}]$ are E[a]=∑P(a)⋅a, which represent the expected local ancestry call for each marker.

### Comparing admixture time estimates

Our final analysis examines the performance of estimating time since admixture using local ancestry calls from phase-free and phase-based LAI. For phase-free LAI, we applied PAPI (Avadhanam and Williams 2022), a tool for inferring parental admixture proportions and times since admixture from unphased local ancestry calls. PAPI produces two estimates of admixture time, one for each parent of the admixed sample, which we average to obtain a single admixture time estimate (${\hat{T}}_{\mathrm{unphased}}$).

To estimate admixture times from phased local ancestry calls, we first grouped together the tracts of Morgan length $x_{i,a}$ corresponding to each ancestry a∈{0,1}, where $0\leq i<N_{a}$, and $N_{a}$ is the number of tracts with ancestry *a*. We then computed four statistics:


(1) $${\hat{\lambda }}_{a}=\frac{N_{a}-m_{a}}{\sum_{i}x_{i,a}}$$

and


(2) $${\hat{\;p}}_{a}=\frac{\sum_{i}x_{i,a}}{\sum_{a^{\prime }}\sum_{i}x_{i,a^{\prime }}}.$$

Here, ${\hat{\lambda }}_{a}$ is the estimated exponential rate parameter for switching from ancestry *a* to the opposite ancestry (switching from a=0 to a=1, for example), and ${\hat{\;p}}_{a}$ is the focal individual’s estimated ancestry fraction from population *a*. The $m_{a}$ term denotes the number of tracts of ancestry *a* that occur at the end of a chromosome (so $m_{a}\leq 22$) and the $-m_{a}$ factor in the numerator of Equation (1) accounts for the fact that a chromosome end prematurely cuts off a tract before the next crossover is observed (Caballero *et al.* 2019). Note that the denominator of Equation (2) is always twice the length of the genome.

Next, we applied PAPI’s internal model for estimating admixture time to these haploid tracts. PAPI assumes that the observed local ancestry tracts are generated by transmitted crossovers within a pedigree that includes $2^{T}$ unadmixed founders of different ancestries. Further, PAPI’s model treats these founder haplotypes as a pool, where the observed haplotypes after *T* generations of crossovers are generated by a Markovian path that switches to a random haplotype in the pool at rate *T* per Morgan. Under this model, the rate of between-ancestry crossovers (or switches) from ancestry *a* to ancestry (1−a) is approximately the overall switch rate *T* times a factor equal to the proportion of haplotypes with ancestry (1−a) in the founder pool (Gravel 2012; Avadhanam and Williams 2022). A reasonable estimate of that proportion is simply the proportion of this ancestry in the admixed individual being analyzed, so:


(3) $$\lambda_{a}=p_{\left(1-a\right)}T.$$

Solving this for *T* gives two estimates of admixture time, one for a=0 and one for a=1: $\hat{T}=\frac{{\hat{\lambda }}_{0}}{{\hat{\;p}}_{1}}$ and $\hat{T}=\frac{{\hat{\lambda }}_{1}}{{\hat{\;p}}_{0}}$. We combined these by simply averaging them, and our estimate of admixture time from phased LAI is thus ${\hat{T}}_{\;phased}=\frac{1}{2}(\frac{{\hat{\lambda }}_{0}}{{\hat{\;p}}_{1}}+\frac{{\hat{\lambda }}_{1}}{{\hat{\;p}}_{0}})$.

## Results

We measured LAI accuracy for two phase-free methods—HAPMIX and LAMP-LD—and two phase-based methods—RFMix and FLARE—across every combination of four input parameters: T∈{5,6,7}, p∈{0.2,0.5,0.8}, phasing sample size (small, medium, and large), and panel phasing strategy (default, reference, and rephase) (see Materials and methods). We further examined a wider range of times since admixture by considering T∈{5,10,15}. Prior to these LAI analyses, we calculated switch error rates in the phased data in order to investigate the sensitivity of phase quality to the parameters; a priori we expected only phasing sample size to have a strong effect on quality. Moreover, to better understand the impact of phase on LAI, we examined the performance of each method using perfectly phased data and also applied a phase correction algorithm that analyzes local ancestry calls (see below). Finally, we estimated the impact of LAI accuracy on downstream admixture time estimates using LAI tracts from one phase-free method (HAPMIX) and one phase-based method (FLARE).

### Phasing sample size and other simulation parameters affect switch error rates

**Fig. 2.**
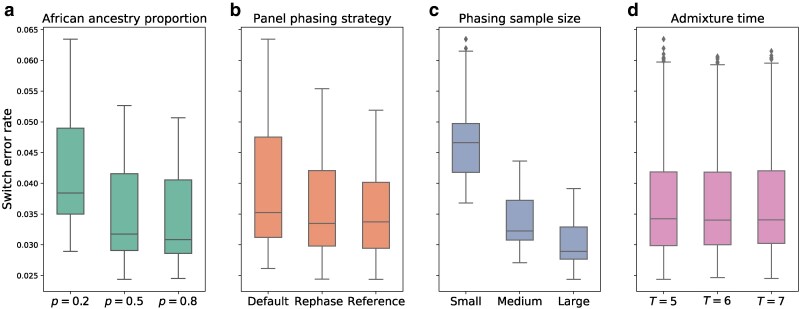
Switch error rates depend on phasing sample size and several simulation parameters. Switch error rates plotted against a) proportion of African ancestry *p*, b) panel phasing strategy, c) phasing sample size, and d) admixture time *T* (i.e. generations since admixture). Each panel includes data points for all values of the other variables. Boxplot lengths represent the inter-quartile range (IQR) and whiskers extend up to 1.5×IQR.

After phasing with SHAPEIT4, we measured switch error rates in the simulated samples for every setting of *T*, *p*, sample size, and panel phasing strategy (Fig. 2, Supplementary Fig. S1). Overall, the variable with the largest impact on phase quality is sample size. Regardless of strategy or simulation setting, jointly phasing the simulated individuals with the largest possible set of accompanying samples produces the lowest switch error rates, as is commonly seen in phasing analyses (Browning and Browning 2011; Fig. 2c). Averaged across all settings of *T*, *p*, and phasing strategy, the switch error rates for the small, medium, and large phasing sample sizes are 4.65%, 3.37%, and 3.01%, respectively. The relative improvement obtained by moving from the small (N=580) to medium (N=2,890) sample size is considerably greater (27.5%) than that of moving from the medium to large (N=5,780) sample size (10.7%). This is again consistent with prior studies: phase quality improves dramatically when increasing from smaller sample sizes, but shows a trend of diminishing returns in larger samples (Browning and Browning 2011).

As may be expected, phase quality is also sensitive to panel phasing strategy (see Fig. 2b, Supplementary Fig. S2): the average switch error rate is lowest for the reference phasing strategy at 3.52%, followed by the rephase strategy at 3.61%, and default phasing at 3.90%. This indicates that including unadmixed reference haplotypes when phasing—either by pooling them with the admixed individuals as in the rephase approach, or conditioning on them as in reference phasing—improves phase quality markedly. However, the effect may be due to an increase in effective phasing sample size and not specifically because we included these unadmixed reference haplotypes.

In turn, phasing simulated individuals with a larger proportion of African ancestry (*p*) yields improved phase, with the lowest switch error rate of 3.40% occurring when p=0.8, followed by 3.48% when p=0.5, and 4.15% when p=0.2 (see Fig. 2a, Supplementary Fig. S3). A possible explanation is that the admixed haplotypes simulated with p=0.8 may be more similar to the admixed haplotypes from the PAGE dataset that they are pooled with—the PAGE per-person average is p=0.71 (Avadhanam and Williams 2022). Finally, the switch error rates show effectively no dependence on the time since admixture *T*, with only slight variation that may be driven by statistical noise (3.68%, 3.67%, and 3.69%, respectively, for T=5, 6, and 7; Fig. 2d).

### Phase-free LAI is more accurate than phase-based LAI for recent admixture and a range of sample sizes

**Fig. 3.**
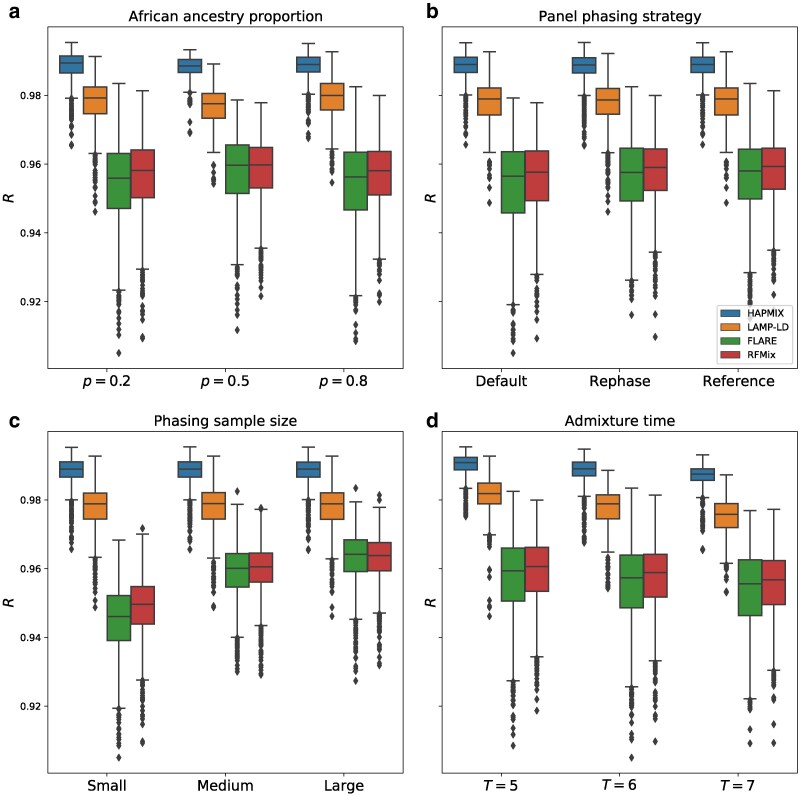
Performance of LAI methods across all simulation parameters. Correlations *R* between inferred and true local ancestry assignments for all LAI methods plotted against a) proportion of African ancestry *p*, b) panel phasing strategy, c) phasing sample size, and d) admixture time *T* (i.e. generations since admixture). Each panel includes data points from all other simulation variables. Boxplot lengths represent the IQR and whiskers extend up to 1.5×IQR.

A central question in our analysis is whether phase-free LAI is more accurate than phase-based LAI given the phase quality our data provided. We find that both phase-free methods (HAPMIX and LAMP-LD) are substantially more accurate than phase-based methods (RFMix and FLARE), even when the latter receive the highest-quality phased data (Fig. 3, Supplementary Fig. S4). In particular, HAPMIX outperforms all other methods in all parameter settings, with an average *R* of 0.988 (range 0.965–0.995); the average *R* of LAMP-LD is slightly lower at 0.978 (range 0.946–0.993). The corresponding *R* values for the phase-based methods are 0.956 for FLARE (range 0.905–0.983) and 0.957 for RFMix (range 0.909–0.981).

A further advantage of using phase-free methods is that they ignore the phase of the target samples by design and are therefore robust to the impact of phasing-specific variables (notably, we input the same data to all tools, but erased the phase for the phase-free methods). In particular, HAPMIX and LAMP-LD show an average *R* that is identical (R=0.988 and 0.978, respectively) across all phasing sample sizes and phasing strategies.

**Fig. 4.**
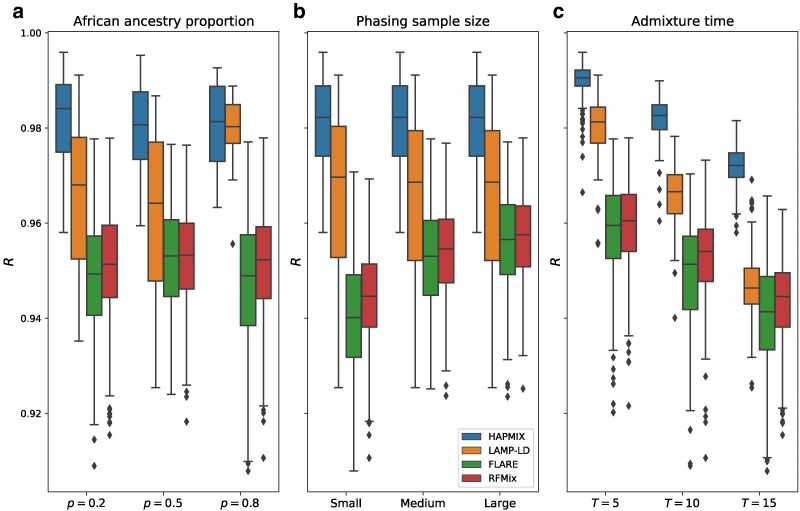
Performance of LAI methods for larger values of *T* using the reference phasing strategy. Each panel shows boxplots of correlations (*R*) between inferred and true local ancestry assignments across different simulation settings of a) proportion of African ancestry *p*, b) phasing sample size, and c) admixture time *T* (i.e. generations since admixture). Boxplot lengths represent the IQR and whiskers extend up to 1.5×IQR.

To assess the generalizability of these findings, we repeated these analyses for simulated admixture times T∈{5,10,15} while applying only the reference phasing strategy (Fig. 4). The performances of all methods diminish as *T* increases, which is unsurprising because local ancestry tract lengths decrease as *T* increases. Notably, LAMP-LD’s accuracy drops more rapidly for larger *T* than other methods, such that while it outperforms the phase-based methods in all settings, when T=15, it is only slightly more accurate at mean R=0.947 versus R=0.940 for FLARE and R=0.944 for RFMix. In turn, RFMix and FLARE have very similar accuracy when T=5 (mean R=0.959 and 0.958, respectively), but RFMix’s relative performance increases for larger *T*, with an accuracy that differs from FLARE’s more noticeably at T=15 (mean R=0.943 and 0.940, respectively). Overall, HAPMIX outperforms all methods by R≥0.03 for all simulated settings of *T*, highlighting the effectiveness of its model that analyzes data free of switch errors.

### Phasing settings and demographic parameters impact LAI performance

As expected for phase-based LAI approaches, RFMix and FLARE are both sensitive to phasing sample size (Fig. 3c, Supplementary Fig. S4), with RFMix performing identically to FLARE for medium (R=0.959) and large (R=0.963) sample sizes, but outperforming FLARE when the sample size is low (R=0.949 and 0.945, respectively). Both methods underperform relative to the phase-free methods, as noted above.

With phase-based LAI, we find that the reference and rephase strategies work equally well and are more accurate than the default strategy (Fig. 3b, Supplementary Fig. S4). Specifically, RFMix has an average accuracy of R=0.958 for both the reference and rephase approaches (identical up to three significant figures) and R=0.956 for the default strategy. FLARE has an average R=0.956 for the reference and rephase strategies, and an *R* value of 0.954 with the default approach. When the input phasing sample sizes are small, the improvements of reference and rephase strategies (R=0.951 and 0.950, respectively, for RFMix and 0.947 for both strategies using FLARE) over the default phasing strategy (R=0.946 and 0.941 for RFMix and FLARE, respectively) are more substantial.

All LAI methods that we examined are relatively insensitive to African admixture proportion *p*. For instance, the phase-based methods RFMix and FLARE have virtually identical (up to three significant figures) average *R* of 0.957 and 0.954, respectively, at p=0.2 and p=0.8 , and a slightly higher *R* of 0.959 and 0.958, respectively, when p=0.5 (Fig. 3a, Supplementary Fig. S4). LAMP-LD has a drop in accuracy between p=0.2 and p=0.5 , from 0.978 to 0.976, and an increase in *R* to 0.979 when p=0.8. Overall, HAPMIX is the most robust to changes in *p*, staying consistently more accurate than the rest at R=0.988 (identically up to three significant figures) for all values of *p*.

In contrast to *p*, all methods show a similar and clear trend of decreasing accuracy as *T* increases (Figs. 3d, 4, Supplementary Fig. S4). More specifically, when moving from T=5 to T=15, the methods’ mean *R* values decrease by 0.017 for HAPMIX, 0.033 for LAMP-LD, 0.018 for FLARE, and 0.016 for RFMix. This is likely due to the increased number of ancestral recombinations for larger values of *T* that produce shorter local ancestry tracts. As shorter tracts have fewer SNPs than longer ones, they have lower informativeness, making LAI more challenging. Furthermore, calling local ancestry at tract boundaries is difficult, and a larger number of such boundaries may also contribute to the reduction in accuracy. Here, as with admixture proportion, HAPMIX remains the most accurate LAI method across the range of admixture times we tested.

### HAPMIX outperforms phase-based methods even when simulated data is free of switch errors

**Fig. 5.**
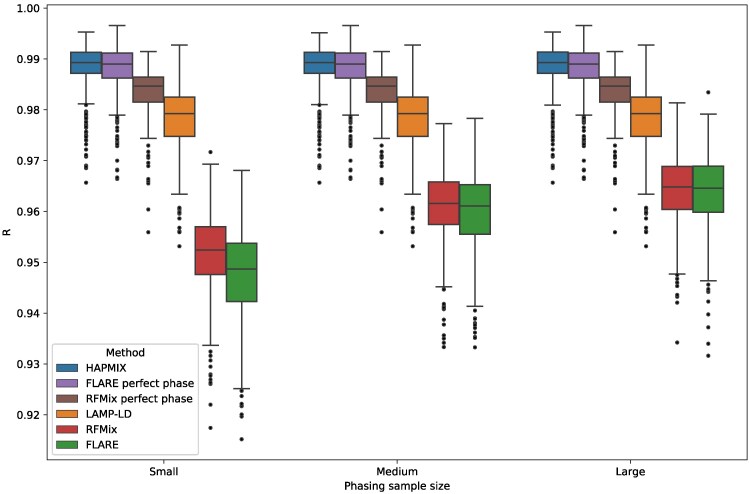
HAPMIX outperforms phase-based LAI even when perfectly phased data is available. Plot shows correlations *R* between the inferred and true local ancestry assignments by phasing sample size, including results for FLARE and RFMix when analyzing perfectly phased data (as produced by the simulation pipeline). Each panel includes data points from all other simulation variables (with T∈{5,6,7}). Boxplot lengths represent the IQR and whiskers extend up to 1.5×IQR.

To further characterize the effect of phase on LAI, we ran each method on the perfectly phased data produced by our simulation pipeline. Strikingly, HAPMIX outperformed all other methods even in this limiting case, with mean R=0.989 (Fig. 5). Even so, FLARE’s performance with mean R=0.988 is on par with that of HAPMIX, and between the phase-based methods, FLARE better exploits the lack of switch errors in these data (RFMix’s mean R=0.983). Both phase-based methods outperformed LAMP-LD’s mean R=0.978 by a considerable margin. This is consistent with another benchmarking study that analyzed perfectly phased two-way admixed samples and found that RFMix has higher accuracy than LAMP-LD (Schubert *et al.* 2020). Yet, it contrasts with our results using statistically phased data (Figs. 3 and 4)—highlighting the importance of analyzing realistically phased data to test LAI methods.

Taken together, our results suggest that HAPMIX’s underlying model can produce the most accurate diploid calls regardless of phase quality or sample demography. It is surprising that HAPMIX performs better than the phase-based methods even when they are given perfect phase. There could be many reasons for this—the modeling approaches differ markedly between HAPMIX (a generative model) and RFMix (a discriminative one); and HAPMIX and FLARE, despite being similar in many respects, each implement different extensions of the Li and Stephens model (for example, HAPMIX has a mis-copying parameter that allows a small probability of copying from a haplotype of a different ancestry than the one represented in the hidden state). In general, the methods differ with respect to parameterization, and HAPMIX could be better tuned for the settings we investigated; there are also compute time versus accuracy tradeoffs—HAPMIX is more accurate but considerably slower than other methods we tested.

We also applied TRACTOR’s phase correction algorithm (Atkinson *et al.* 2021), which rephases input data by detecting local ancestry-based switch errors, i.e. nearby positions where the ancestries of the two haplotypes switch. RFMix’s performance after applying TRACTOR is modestly improved, with R=0.960 using the corrected haplotypes versus R=0.957 with the original data (averaged over all simulation parameters). With the exception of the default panel phasing strategy, the performance improves across all sample sizes, demographic parameters, and phasing conditions (Supplementary Fig. S5). Despite this, the accuracy of the phase-corrected LAI calls remain much lower than that of the HAPMIX calls. For example, the average *R* for HAPMIX is 0.988, which is still a 2.92% increase over TRACTOR-corrected RFMix (R=0.960).

### Admixture time estimates using phase-based LAI are strongly biased

**Fig. 6.**
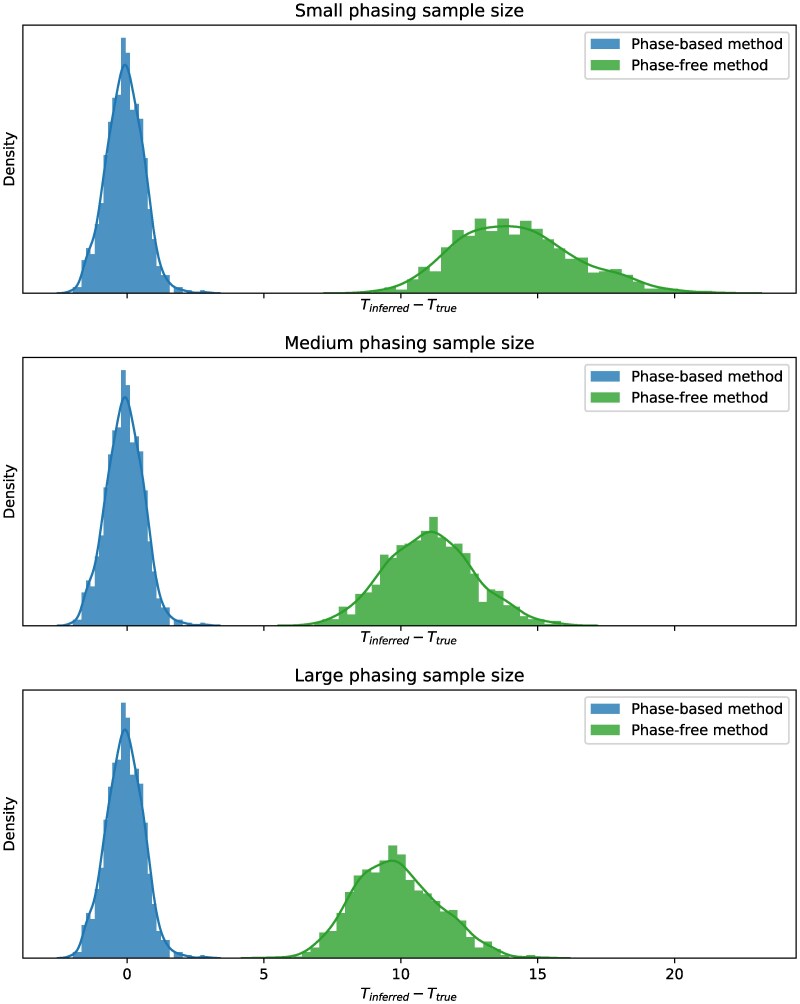
Deviations of admixture time estimates using local ancestry calls from HAPMIX and FLARE. Plots depict histograms of the deviations of admixture time estimates for individual samples using a phase-free method (applying PAPI to the output of HAPMIX) and a phase-based method (applying Equation (3) to the output of FLARE) for small (top), medium (middle), and large (bottom) phasing sample sizes. Data points are pooled across all other simulation variables.

To characterize the impact of LAI accuracy on downstream applications, we calculated admixture time estimates using the output of both FLARE and HAPMIX. Because HAPMIX produces unphased local ancestry calls, we provided these as input to PAPI (Avadhanam and Williams 2022) and report its time estimates; we also applied a model that parallels that of PAPI to FLARE’s tracts (see Materials and methods). Figure 6 plots the deviations of the admixture time estimates from the ground truth ($T_{\mathrm{inferred}}-T_{\mathrm{true}}$) for the reference phasing strategy data and for each phasing sample size. Strikingly, estimates using HAPMIX’s calls are virtually unbiased, while the FLARE-based estimates are strongly upwardly biased, even when the phase quality is high. Specifically, the average deviation values using FLARE segments are 14.2, 11.1, and 9.84 for small, medium, and large sample sizes, respectively. (For context, FLARE also internally estimates admixture time and these values are similarly upwardly biased, with an average deviation of 9.61 for the large sample size and reference phasing strategy setting, considering $T_{\mathrm{true}}\in \{5,10,15\}$.) In contrast, HAPMIX has a slight downward bias of −0.0909 for all sample sizes (as a phase-free LAI method, HAPMIX’s results are expected to remain unchanged with respect to phasing sample size).

## Discussion

In this work, we analyzed the performance of four state-of-the-art LAI methods and found that despite large sample sizes and good quality phase, phase-based methods do not perform as well as phase-free ones in terms of call accuracy of unphased ancestry states (Fig. 3). Furthermore, unphased LAI remains more effective even after applying TRACTOR’s phase correction algorithm, which identifies likely switch errors from changes in the local ancestry on both haplotypes of an individual (Supplementary Fig. S5). Even so, use of perfectly phased data greatly improves the phase-based methods’ performance, with FLARE’s accuracies being only slightly inferior to those of HAPMIX in this case (Fig. 5).

Based on our analyses, the most important factor that affects phase quality in admixed individuals is sample size (Fig. 2). Moreover, for a fixed sample size, we found empirically that it is best to include the unadmixed reference haplotypes when phasing, with a slight improvement by conditioning on the pre-phased 1,000 G data (using, e.g. SHAPEIT's --reference or Beagle’s ref option Browning *et al.* 2021) rather than by pooling them with the admixed individuals (as in the rephasing strategy). That is, there is an improvement in phase quality alone by including these reference haplotypes—perhaps because they increase the sample size. Additionally, conditioning on reference haplotypes or rephasing them makes the target individuals’ phase more consistent with these panels and is therefore recommended for LAI and other downstream processes (such as imputation) that utilize reference panels.

A combination of both switch errors breaking up ancestry tracts and reduced accuracy of phase-based LAI methods can substantially impact downstream analyses that leverage these data, such as estimating admixture times. Our findings show that even with good phase quality (i.e. phasing sample sizes of 5,810 individuals), admixture time estimates derived from FLARE’s output are biased by more than an order of magnitude relative to those based on HAPMIX calls (Fig. 6). Indeed, concern over phase-based LAI quality motivated us to develop PAPI for estimating time since admixture and parent ancestry proportions using unphased local ancestry calls (Avadhanam and Williams 2022). Because of these issues, many estimates of time since admixture use trio-phased data (Gravel 2012), but given the quality of phase-free methods, tools such as PAPI can leverage the more abundant nontrio samples to perform even sample-specific admixture time estimates.

The difference in accuracy between phase-based and phase-free LAI is substantial enough that careful deliberation on whether to apply phase-based LAI may be warranted, depending on the setting. This may especially be true when analyzing nonmodel organisms where sample sizes are sometimes small and, therefore, phase quality low. Our results suggest that HAPMIX, in particular, is considerably more accurate than the other methods in all parameter settings we considered (see Results), making it a clear choice when high-quality LAI calls are required but high-quality phase is unavailable. Of course, a key limitation of HAPMIX is that it applies only to two-way admixed samples; yet LAMP-LD is a competitive alternative (R=0.978 on average versus HAPMIX’s R=0.988) that can perform LAI in multiway admixed samples. We note that our primary metric is the per-site correlation of the true local ancestry with each method’s local ancestry calls, where we calculated the expected call per-site for HAPMIX since its output is probabilistic. HAPMIX’s performance may benefit from this choice of metric, but this reflects its more informative probabilistic output—information that can be incorporated in downstream applications. When considering which LAI method to apply, another important factor is runtime. While phase-free methods may be slower than phase-based ones, it is worth noting that the latter require the input to be phased, which can be a computationally intensive step.

There are notable limitations to this study. The sample sizes we used yield good but far less accurate phase than is available with biobank-scale data (Delaneau *et al.* 2019; Browning *et al.* 2021), and phase-based LAI performance may be competitive with phase-free methods given such well-phased data. Indeed, we found that RFMix outperforms LAMP-LD when using perfectly phased data (mirroring the results of a previous study Schubert *et al.* 2020), while in our statistically phased samples, LAMP-LD performs better, consistent with earlier work (Dias-Alves *et al.* 2018; Fig. 5). Further, we focused on two-way admixed samples, examining the archetypal case of admixture between relatively divergent populations (Africans and Europeans) where we can expect the highest quality LAI. In that regard, our work can be viewed as a best-case empirical analysis for sub-biobank-scale data, and demonstrates that challenges for phase-based methods apply even in such settings. Our simulated admixed samples are generated from a subset of the unadmixed 1,000 G individuals, and we excluded these individuals from the LAI reference panels, reducing their size. The size of these panels typically impacts LAI quality, and previous work found that MOASIC is more robust to small reference panels than both RFMix and FLARE (Browning *et al.* 2023). Thus, the phase-based method MOASIC may outperform these methods in our analyses. Additionally, we considered investigating three-way admixture such as those of Latin American populations, but due to a lack of both unadmixed Indigenous American reference panel data and three-way admixed individuals where all samples are genotyped on substantial numbers of overlapping sites, we excluded this analysis from the current study (data for admixed samples are required to realistically phase the simulated individuals). Finally, our analysis considered only recent admixture times (T≤15), and future work studying an expanded range of such times and multiway admixture would be valuable.

This work highlights important concerns that can arise in LAI from phase quality issues—even when the phasing is done with several thousand genotyped samples. Future LAI methods development could focus on new phase-free approaches or on phase-based methods that internally model and correct the switch errors most likely to impact accuracy, such as those that change the ancestry of the underlying haplotypes. An effective approach would be similar to the corrections TRACTOR makes (while having the advantage of access to the complete internal model) and would deviate from current approaches (Maples *et al.* 2013). Our findings underscore the importance of designing studies that apply LAI carefully, with due consideration to factors such as phase quality, utilization of reference panels, choice of LAI method, and admixture demography. Most crucially, we demonstrate that the phase quality of the admixed samples has a large impact on LAI accuracy for phase-based methods, an issue implicitly mitigated by phase-free LAI methods. Finally, while the current study analyzes human data, the issues we highlight here are particularly relevant in settings where high-quality reference datasets may not be readily available, such as for nonmodel organisms.

## Supplementary Material

jkaf122_Supplementary_Data

## Data Availability

Genotype data for BioMe Biobank subset of the PAGE II dataset are available (dbGaP:phs000925.v1.p1), and the phased 1,000 Genomes data used for local ancestry inference is publicly available at https://ftp-trace.ncbi.nih.gov/1000genomes/ftp/release/20130502/. The simulated genotype data supporting the current study have not been deposited in a public repository but can be reproduced using scripts distributed with PAPI, Ped-sim (https://github.com/williamslab/ped-sim) and admix-simu (https://github.com/williamslab/admix-simu). Supplemental material available at G3 online.
